# ﻿Three new species of *Fusarium* (Nectriaceae, Hypocreales) isolated from Eastern Cape dairy pastures in South Africa

**DOI:** 10.3897/mycokeys.115.148914

**Published:** 2025-03-20

**Authors:** Claudette Dewing, Cobus M. Visagie, Emma T. Steenkamp, Brenda D. Wingfield, Neriman Yilmaz

**Affiliations:** 1 Department of Biochemistry, Genetics and Microbiology, Forestry and Agricultural Biotechnology Institute (FABI), Faculty of Natural and Agricultural Sciences, University of Pretoria, Pretoria, South Africa University of Pretoria Pretoria South Africa

**Keywords:** *Fusariumcamptoceras*, GCPSR, molecular phylogenetics, morphology, mycotoxins

## Abstract

A survey of the fungal diversity associated with mixed pastures from Eastern Cape dairy farms in South Africa led to the isolation of 155 *Fusarium* strains that belong to the *Fusariumincarnatum-equiseti* species complex (FIESC). Using single and multigene phylogenies based on partial sequences of the translation elongation factor 1-alpha (*TEF*), calmodulin (*CaM*), and the partial RNA polymerase second largest subunit (*RPB2*) genes, we identified 11 species. They included *F.brevicaudatum*, *F.clavus*, *F.coffeatum*, *F.croceum*, *F.goeppertmayerae*, and *F.heslopiae*, with five species that were found to be new. Based on morphological and phylogenetic data, three new species are formally described here as *F.cumulatum*, *F.mariecurieae*, and *F.pascuum*. We also provided a description for *F.goeppertmayerae*, as the authors who identified and named this species did not include one. We have chosen to not describe the remaining species, as our cultures lack proper morphological structure development. This study shows that mixed pastures harbour a diverse range of *Fusarium* species and highlights the need for further studies into their potential to impact animal health.

## ﻿Introduction

Well-maintained pastures are important for promoting the well-being of cattle, as they directly impact the animals’ nutrition, overall health and productivity (Marais 2001; Neal et al. 2007; Botha et al. 2008a, 2008b; Doxey 2014; van der Colf et al. 2015). Several grass species from Poaceae are used for grazing worldwide (Charlton and Stewart 1999; Lowe 2009; Truter et al. 2015). In South Africa the preferred species are *Cenchrusclandestinus* (formerly known as *Pennisetumclandestinum*; kikuyu), *Loliummultiflorum* (annual ryegrass) and *L.perenne* (perennial ryegrass) (Truter et al. 2015). Despite the obvious nutritional value of pastures, they can also pose health risks to grazing animals under certain circumstances, e.g., kikuyu poisoning in cattle (Bryson and Newsholme 1978; Newsholme et al. 1983; Wong et al. 1987; Bourke 2007; Ryley et al. 2007; Botha et al. 2014) or when the growth of mycotoxigenic fungi leads to mycotoxin build-up in pastures (Golinski et al. 2003; Driehuis et al. 2008; Skládanka et al. 2011; Penagos-Tabares et al. 2021).

Among the various factors that could affect the health of grazing cattle, *Fusarium* species and their mycotoxins pose a significant risk. The genus *Fusarium* comprises a diverse group of filamentous fungi that play significant roles in various ecological and agricultural contexts because of the range of lifestyles they exhibit, including saprotrophic and endophytic modes, often associated with various grass hosts (Leslie et al. 2004; Bentley et al. 2007; Summerell et al. 2010; Nor Azliza et al. 2014; Laurence et al. 2016). Some *Fusarium* species are also well-known pathogens to animals, humans and plants, and are important producers of mycotoxins (Desjardins 2006; Leslie and Summerell 2006; O’Donnell et al. 2013; Gallo et al. 2022). *Fusarium* mycotoxins that are particularly important from a toxicological standpoint include deoxynivalenol (Reed and Moore 2009; Skládanka et al. 2011; Burkin and Kononenko 2015; Penagos-Tabares et al. 2021), fumonisins (Reed and Moore 2009; Gott et al. 2017) and zearalenone (di Menna et al. 1987; Reed et al. 2004; Reed and Moore 2009; Skládanka et al. 2011; Burkin and Kononenko 2015; Nichea et al. 2015; Penagos-Tabares et al. 2021). These toxins can cause severe health issues in animals, including abnormal foetal development, disruptions in cell division and membrane function, reduced feed intake leading to body weight loss, fertility problems, immunosuppression, inhibition of protein synthesis, impaired mitochondrial function and, in severe cases, death (Trenholm et al. 1985; Weaver et al. 1986; Smith et al. 1990; Edrington et al. 1995; Moretti et al. 2013; Eskola et al. 2018; Aranega and Oliveira 2022). Furthermore, certain *Fusarium* species and their associated mycotoxins have been suggested as potential contributors to kikuyu poisoning cases in South Africa and Australia (Ryley et al. 2007; Botha et al. 2014). However, this connection remains hypothetical and unconfirmed due to limited availability of supporting data.

In 2020, cattle in the Eastern Cape province of South Africa showed symptoms of facial eczema, a type of hepatogenous photosensitivity caused by the mycotoxin sporidesmin A, produced by the fungus *Pseudopithomyceschartarum* (= *Pithomyceschartarum*) (Brook 1963; di Menna et al. 1970; Marasas and Schumann 1972; Kellerman et al. 1980; Kellerman and Coetzer 1985; Davis et al. 2021). A fungal survey was subsequently conducted at 14 dairy farms to first determine whether *P.chartarum* was present in affected pastures and second to identify other culturable fungi that may also be present (Dewing et al. 2025). The survey revealed *Fusarium* as the most commonly isolated genus, particularly species within the *Fusariumincarnatum-equiseti* species complex (FIESC). However, some strains in the complex could not be satisfactorily identified to species level. Here we report on the FIESC species present in Eastern Cape dairy pastures and describe three new species using macro- and micro-morphological characterisation with the support of multigene phylogenetic approaches. We also supply *F.goeppertmayerae* with the necessary macro- and micro-morphological characterisation information, as this was not supplied by Tan and Shivas (2023), who identified and named this species.

## ﻿Materials and methods

### ﻿Sampling and isolations

A total of 95 mixed pasture grass samples (primarily a mixture of kikuyu and ryegrass) were collected from 14 dairy farms in the Eastern Cape province of South Africa in May 2020, with a specific focus on identifying *Fusarium* species (Table 1). These pastures were potentially associated with a facial eczema outbreak in cattle. Plant material was cut into small pieces (±4 mm) and plated onto potato dextrose agar (PDA; Becton, Dickinson and Company (BD), Franklin Lakes, USA) and water agar (WA). Both were supplemented with chloramphenicol (100 ppm). The plates were incubated for 7–10 d at 25 °C and checked regularly for fungal growth. Colonies were transferred to pure cultures onto ¼PDA supplemented with chloramphenicol (100 ppm). Single spore cultures were prepared for all *Fusarium* strains following Leslie and Summerell (2006). Strains were accessioned and preserved in cryovials containing 10% glycerol and stored at -80 °C in the Applied Mycology working culture collection (CN) housed at the Forestry and Agricultural Biotechnology Institute (FABI) at the University of Pretoria, South Africa. Additionally, representative strains were deposited in the culture collections of FABI (Collection Mike Wingfield (CMW) and Collection Mike Wingfield at Innovation Africa (CMW-IA)) and the Westerdijk Fungal Biodiversity Institute (CBS) in Utrecht, the Netherlands (Table 1).

**Table 1.** *Fusarium* strains from the *Fusariumincarnatum-equiseti* species complex (FIESC) isolated from mixed dairy pastures from the Eastern Cape, South Africa.

Species	Strain	*TEF*	*CaM*	*RPB1*	*RPB2*
***Camptoceras*-clade (FIESC; Han et al. (2023) = orginally the FCAMSC)**					
*F.pascuum* sp. nov.	CMW-IA 003320 = CMW 61364 = CN056A8	OR670986	OR669177	PP187127	PP235233
*F.pascuum* sp. nov.	CMW 58649 = CN070E7	PP187098	—	PP187129	PQ467747
*F.pascuum* sp. nov.	CMW 58650 = CN070F7	OR670991	OR669181	PP187132	PP158160
*F.pascuum* sp. nov.	CMW-IA 002133 = CMW 60931 = CN070I3	PP187102	PP187122	—	PQ467748
*F.pascuum* sp. nov.	CMW 58651 = CN070I4	OR671007	OR669194	PP187144	PQ467749
*F.pascuum* sp. nov.	CMW 58652 = CN071B8	OR671025	OR669210	PP187152	PP158176
*F.pascuum* sp. nov.	CBS 151772 = CMW 58653 = CN159G4 = CN071C4	OR671027	OR669212	PP187155	PP158178
*F.pascuum* sp. nov.	CMW 58654 = CN071D3	OR671033	OR669216	PP187158	PQ467750
*F.pascuum* sp. nov.	CMW 58655 = CN071E9	OR671039	OR669221	PP187161	PP158183
*F.pascuum* sp. nov.	CMW 58656 = CN071F9	OR671044	OR669225	PP187166	PP235238
*F.pascuum* sp. nov.	CMW 58657 = CN071G8	OR671051	OR669229	PP187168	PQ467751
*F.pascuum* sp. nov.	CMW 58658 = CN071I3	OR671057	OR669233	PP187173	PQ467752
*F.pascuum* sp. nov.	CMW 58659 = CN071I5	OR671058	OR669234	PP187175	PP158189
*F.pascuum* sp. nov.	CMW 58660 = CN071I9	OR671061	OR669237	PP187179	PQ467753
*F.pascuum* sp. nov.	CMW 58661 = CN072A1	OR671062	OR669238	PP187180	PP158191
*F.pascuum* sp. nov.	CMW 58662 = CN104D6	OR671090	OR669265	PP187190	PP158199
*F.pascuum* sp. nov.	CMW 58663 = CN104D7	OR671091	OR669266	PP187191	PQ467754
***Equiseti*-clade (FIESC)**					
*F.brevicaudatum*	CMW-IA 003335 = CMW 61379 = CN071C6	OR671028	—	—	—
*F.brevicaudatum*	CMW-IA 003765 = CMW 61537 = CN110E5	OR671114	—	—	—
*F.clavus*	CMW-IA 001930 = CMW 60748 = CN041D2	OR670983	—	—	—
*F.clavus*	CMW-IA 003330 = CMW 61374 = CN070H4	OR671001	OR669189	—	—
*F.clavus*	CN070H7	PP187101	PP187121	—	—
*F.clavus*	CN070I8	OR671010	OR669197	—	PP158167
*F.clavus*	CN071A3	OR671013	OR669200	—	—
*F.clavus*	CN071A4	OR671014	OR669201	—	—
*F.clavus*	CN071B3	OR671020	OR669205	—	—
*F.clavus*	CN071B4	OR671021	OR669206	—	PP158174
*F.clavus*	CN071D1	OR671031	OR669215	—	—
*F.clavus*	CN071D2	OR671032	—	—	—
*F.clavus*	CN071D8	OR671034	OR669217	—	—
*F.clavus*	CN071E1	OR671035	OR669218	—	—
*F.clavus*	CN071E2	OR671036	—	—	PP158180
*F.clavus*	CN071G5	OR671049	OR669227	—	PP158187
*F.clavus*	CN071G6	OR671050	OR669228	—	—
*F.clavus*	CN071H6	OR671054	-	—	—
*F.clavus*	CN072A6	OR671066	OR669241	—	PP158193
*F.clavus*	CN072A9	OR671069	OR669244	—	PP158195
*F.clavus*	CN072E4	OR671076	OR669251	—	PP158196
*F.croceum*	CMW-IA 001923 = CMW 60732 = CN040I7	OR670982	—	—	—
*F.croceum*	CMW-IA 001934 = CMW 60752 = CN041D9	OR670984	—	—	—
*F.croceum*	CN048C3	OR670985	—	—	—
*F.croceum*	CN070F6	OR670990	—	—	—
*F.croceum*	CMW-IA 003326 = CMW 61370 = CN070F8	OR670992	—	—	—
*F.croceum*	CN070F9	PP187099	—	—	PQ467755
*F.croceum*	CN070G4	OR670995	OR669184	PP187135	PP158162
*F.croceum*	CN070G6	OR670997	—	PP187137	PQ467756
*F.croceum*	CN070G7	OR670998	OR669186	PP187138	PQ467757
*F.croceum*	CN070H1	OR671000	OR669188	—	—
*F.croceum*	CN070H5	OR671002	OR669190	—	—
*F.croceum*	CN070I1	OR671005	OR669193	PP187143	PQ467758
*F.croceum*	CN070I2	OR671006	—	—	—
*F.croceum*	CN070I5	OR671008	OR669195	PP187145	PP158165
*F.croceum*	CN070I9	OR671011	OR669198	PP187147	PP158168
*F.croceum*	CN071B1	OR671018	OR669203	PP187148	PP158173
*F.croceum*	CN071C7	OR671029	OR669213	PP187156	PQ467759
*F.croceum*	CN071F1	OR671040	OR669222	PP187162	PP158184
*F.croceum*	CN071G1	OR671045	—	—	—
*F.croceum*	CN071G2	OR671046	—	—	—
*F.croceum*	CN071G3	OR671047	—	PP187167	—
*F.croceum*	CN071H3	PP187104	—	PP187170	PQ467760
*F.croceum*	CN071H7	OR671055	OR669231	PP187171	PP158188
*F.croceum*	CN071I4	PP187105	PP187124	PP187174	PP235239
*F.croceum*	CN072A5	OR671065	OR669240	—	—
*F.croceum*	CN072A8	OR671068	OR669243	PP187182	PP158194
*F.croceum*	CN072B1	OR671070	OR669245	PP187183	PQ467761
*F.croceum*	CN072B4	OR671072	OR669247	—	—
*F.croceum*	CN072B8	OR671074	OR669249	—	PP235242
*F.croceum*	CN103E5	OR671077	OR669252	—	—
*F.croceum*	CN103E6	OR671078	OR669253	—	—
*F.croceum*	CN104B9	OR671079	OR669254	—	—
*F.croceum*	CN104C1	OR671080	OR669255	—	—
*F.croceum*	CN104C3	OR671082	OR669257	—	—
*F.croceum*	CN104C4	OR671083	OR669258	—	—
*F.croceum*	CN104C5	OR671084	OR669259	—	—
*F.croceum*	CN104C7	OR671085	OR669260	—	—
*F.croceum*	CN104C9	OR671086	OR669261	—	—
*F.croceum*	CN104D5	OR671089	OR669264	—	—
*F.croceum*	CN104D8	OR671092	OR669267	—	—
*F.croceum*	CN104E4	OR671094	—	—	—
*F.croceum*	CN104E8	OR671095	—	—	—
*F.croceum*	CN106E9	OR671096	—	—	—
*F.croceum*	CN110D4	OR671107	—	—	—
*F.croceum*	CN110D6	OR671109	—	—	—
*F.croceum*	CN110D8	OR671110	—	—	—
*F.croceum*	CN110E1	OR671112	—	—	—
*F.croceum*	CN115A2	PP187108	—	—	—
*F.croceum*	CN115A3	PP187109	—	—	—
*F.croceum*	CN115A4	PP187110	—	—	—
*F.croceum*	CN115B2	PP187111	—	—	—
*F.croceum*	CN115B6	PP187112	—	—	—
*F.croceum*	CN115C8	PP187114	—	—	—
*F.croceum*	CN115D9	PP187117	—	—	—
*F.croceum*	CN119E7	PP187119	—	—	—
*F.cumulatum* sp. nov.	CMW 58686 = CN071B9	OR671026	OR669211	PP187153	PP158177
*F.cumulatum* sp. nov.	CMW 58687 = CN071E5	OR671038	OR669220	PP187160	PP158182
*F.cumulatum* sp. nov.	CMW-IA 002138 = CMW 60936 = CN071G4	OR671048	OR669226	—	PP158186
*F.cumulatum* sp. nov.	CBS 151773 = CMW 58688 = CN104D3	OR671087	OR669262	PP187188	PP158197
*F.heslopiae*	CN071C8	OR671030	OR669214	PP187157	PP158179
***Incarnatum*-clade (FIESC)**					
*F.coffeatum*	CMW-IA 003332 = CMW 61376 = CN071A2	OR671012	OR669199	—	PP158169
*F.coffeatum*	CN071A5	OR671015	OR669202	—	PP158170
*F.coffeatum*	CN071A6	OR671016	—	—	PP158171
*F.coffeatum*	CN071A7	OR671017	—	—	PP158172
*F.coffeatum*	CMW-IA 003334 = CMW 61378 = CN071B5	OR671022	OR669207	—	PP158175
*F.coffeatum*	CMW-IA 003341 = CMW 61385 = CN072A4	OR671064	—	—	—
*F.coffeatum*	CN072A7	OR671067	OR669242	—	—
*F.goeppertmayerae*	CBS 151775 = CMW 58689 = CN040I5	OR670981	OR669176	PP187126	PP158159
*F.goeppertmayerae*	CMW 58690 = CN070F3	OR670988	OR669179	PP187130	PP235234
*F.goeppertmayerae*	CMW 58691 = CN070G8	OR670999	OR669187	PP187139	PP158163
*F.goeppertmayerae*	CMW 58692 = CN070G9	PP187100	PP187120	PP187140	PP235236
*F.goeppertmayerae*	CMW-IA 002132 = CMW 60930 = CN070H9	OR671004	OR669192	PP187142	PP158164
*F.goeppertmayerae*	CMW-IA 003340 = CMW 61384 = CN071H2	OR671053	—	—	—
*F.goeppertmayerae*	CMW 58693 = CN071H8	OR671056	OR669232	PP187172	PQ467762
*F.goeppertmayerae*	CMW 58694 = CN071I6	OR671059	OR669235	PP187176	PP158190
*F.goeppertmayerae*	CMW 58695 = CN071I7	PP187106	PP187125	PP187177	PQ467763
*F.goeppertmayerae*	CMW 58696 = CN071I8	OR671060	OR669236	PP187178	PP235240
*F.goeppertmayerae*	CMW 58697 = CN104D4	OR671088	OR669263	PP187189	PP158198
*F.goeppertmayerae*	CMW 58698 = CN106F2	OR671098	OR669270	PP187194	PP158201
*F.goeppertmayerae*	CMW 58699 = CN106F3	OR671099	OR669271	PP187195	PP235244
*F.goeppertmayerae*	CMW 58700 = CN106F4	OR671100	OR669272	PP187196	PP158202
*F.mariecurieae* sp. nov.	CMW 58664 = CN070E5	OR670987	OR669178	PP187128	PQ467764
*F.mariecurieae* sp. nov.	CMW-IA 002131 = CMW 60929 = CN070F5	OR670989	OR669180	PP187131	—
*F.mariecurieae* sp. nov.	CMW 58665 = CN070G2	OR670994	OR669183	PP187134	PP158161
*F.mariecurieae* sp. nov.	CMW-IA 003328 = CMW 61372 = CN070G5	OR670996	OR669185	PP187136	PP235235
*F.mariecurieae* sp. nov.	CMW 58666 = CN070H6	OR671003	OR669191	PP187141	PQ467765
*F.mariecurieae* sp. nov.	CBS 151774 = CMW 58667 = CN070I7	OR671009	OR669196	PP187146	PP158166
*F.mariecurieae* sp. nov.	CMW 58668 = CN071B2	OR671019	OR669204	PP187149	PQ467746
*F.mariecurieae* sp. nov.	CMW 58669 = CN071C1	PP187103	PP187123	PP187154	PQ467766
*F.mariecurieae* sp. nov.	CMW 58670 = CN071E4	OR671037	OR669219	PP187159	PP158181
*F.mariecurieae* sp. nov.	CMW-IA 002136 = CMW 60934 = CN071F2	OR671041	—	PP187163	PP235237
*F.mariecurieae* sp. nov.	CMW-IA 002137 = CMW 60935 = CN071F3	OR671042	OR669223	PP187164	PQ467767
*F.mariecurieae* sp. nov.	CMW 58671 = CN071F4	OR671043	OR669224	PP187165	PP158185
*F.mariecurieae* sp. nov.	CMW 58672 = CN071H1	OR671052	OR669230	PP187169	PQ467768
*F.mariecurieae* sp. nov.	CBS 152079 = CMW 58673 = CN072A3	OR671063	OR669239	PP187181	PP158192
*F.mariecurieae* sp. nov.	CMW 58674 = CN072B2	OR671071	OR669246	PP187184	PP235241
*F.mariecurieae* sp. nov.	CMW 58675 = CN072B6	OR671073	OR669248	PP187185	PQ467769
*F.mariecurieae* sp. nov.	CMW 58676 = CN072E2	OR671075	OR669250	PP187186	PQ467770
*F.mariecurieae* sp. nov.	CMW 58677 = CN104C2	OR671081	OR669256	PP187187	PP235243
*F.mariecurieae* sp. nov.	CMW 58678 = CN104E1	OR671093	OR669268	PP187192	PQ467771
*F.mariecurieae* sp. nov.	CMW 58679 = CN106F1	OR671097	OR669269	PP187193	PP158200
*F.mariecurieae* sp. nov.	CMW 58680 = CN106F8	PP187107	—	—	—
*F.mariecurieae* sp. nov.	CMW-IA 003763 = CMW 61535 = CN106F9	OR671101	OR669273	PP187197	PP235245
*F.mariecurieae* sp. nov.	CMW 58681 = CN106G1	OR671102	OR669274	PP187198	PP158203
*F.mariecurieae* sp. nov.	CMW 58682 = CN106G2	OR671103	—	PP187199	PQ467772
*F.mariecurieae* sp. nov.	CMW 58683 = CN106G3	OR671104	—	PP187200	—
*F.mariecurieae* sp. nov.	CMW 58684 = CN106G4	OR671105	—	—	—
*F.mariecurieae* sp. nov.	CN106G5	OR671106	OR669275	PP187201	PP235246
*F.mariecurieae* sp. nov.	CMW-IA 003764 = CMW 61536 = CN110D9	OR671111	OR669277	PP187202	PP235247
*F.mariecurieae* sp. nov.	CMW 58685 = CN110E2	OR671113	OR669278	PP187203	PP158204
*F.mariecurieae* sp. nov.	CN115C6	PP187113	—	—	—
*F.mariecurieae* sp. nov.	CN115C9	PP187115	—	—	—
*F.mariecurieae* sp. nov.	CN115D4	PP187116	—	—	—
*F.mariecurieae* sp. nov.	CN115E8	PP187118	—	—	—
*Fusarium*FIESC 27	CMW-IA 003327 = CMW 61371 = CN070G1	OR670993	OR669182	PP187133	PQ276899
*Fusarium* sp. nov. 1	CMW-IA 002134 = CMW 60932 = CN071B6	OR671023	OR669208	PP187150	PQ467773
*Fusarium* sp. nov. 1	CMW-IA 002135 = CMW 60933 = CN071B7	OR671024	OR669209	PP187151	PQ467774

### ﻿DNA extraction, PCR, and sequencing

Genomic DNA was extracted from 7-d-old fungal cultures grown on ¼PDA and incubated at 25 °C, using the PrepMan Ultra Sample Preparation Reagent (Thermo Fisher Scientific, Waltham, USA) following the manufacturer’s instructions. PCR amplification of the translation elongation factor 1-alpha (*TEF*), calmodulin (*CaM*), RNA polymerase largest subunit (*RPB1*) and RNA polymerase second largest subunit (*RPB2*) loci was conducted using primer pairs and thermal cycle conditions as described in Table 2. The PCR reactions were set up in 25 μL volumes using 17.3 µL Milli-Q water (Millipore Corporation, Burlington, USA), 2.5 µL 10 × FastStart Taq PCR reaction buffer containing 20 mM MgCl_2_ (Sigma-Aldrich, Roche Diagnostics, Manheim, Germany), 2.5 µL of 100 mM of each deoxynucleotide (New England Biolabs, Inc., Ipswich, USA), 0.5 µL forward primer (10 µM), 0.5 µL reverse primer (10 µM), 0.5 µL 25 mM MgCl_2_ (Sigma-Aldrich, Roche Diagnostics), 0.2 µL of 5 U/µL FastStart Taq DNA Polymerase (Sigma-Aldrich, Roche Diagnostics, Manheim, Germany), and 1 µL template DNA. PCR products were prepared for sequencing using 2 µL ExoSAP-IT PCR clean-up reagent (1 U/µL FastAP Thermosensitive Alkaline Phosphatase, 20 U/µL Exonuclease I (Thermo Fisher Scientific, Waltham, USA)) and 5 µL PCR product. Sequencing was done bi-directionally using the BigDye terminator sequencing kit v. 3.1 (Applied Biosystems, Foster City, USA) with the same primers used for PCR amplification. Reactions were analysed on an ABI PRISM 3100 DNA sequencer (Applied Biosystems, Foster City, USA). Contigs were assembled and edited in Geneious Prime v. 2019.2.1 (BioMatters Ltd., Auckland, New Zealand). All sequences generated in this study were deposited in GenBank, with accession numbers provided in Table 1.

**Table 2.** Primer pairs and PCR conditions used in this study.

Locus	PCR amplification procedure	Primer	Primer sequence (5’-3’)*	Reference
*TEF*	95 °C 5 min; 35 cycles of 95 °C 45 s, 52 °C 45 s, 72 °C 90 s; 72 °C 8 min; 10 °C soak	EF1	ATGGGTAAGGARGACAAGAC	O’Donnell et al. (1998)
		EF2	GGARGTACCAGTSATCATG	O’Donnell et al. (1998)
*CaM*	94 °C 90 s; 35 cycles of 94 °C 45 s, 50 °C 45 s, 72 °C 1 min; 72 °C 10 min; 10 °C soak	CL1	GARTWCAAGGAGGCCTTCTC	O’Donnell et al. (2000)
		CL2A	TTTTTGCATCATGAGTTGGAC	O’Donnell et al. (2000)
*RPB1*	94 °C 90 s; 5 cycles of 94 °C 45 s, 54 °C 45 s, 72 °C 2 min; 5 cycles of 94 °C 45 s, 53 °C 45 s, 72 °C 2 min; 35 cycles of 94 °C 45 s, 52 °C 45s, 72 °C 2 min; 72 °C 10 min; 10 °C soak	Fa	CAYAARGARTCYATGATGGGWC	Hofstetter et al. (2007)
		R8	CAATGAGACCTTCTCGACCAGC	O’Donnell et al. (2010)
	94 °C 90 s; 5 cycles of 94 °C 45 s, 56 °C 45 s, 72 °C 2 min; 5 cycles of 94 °C 45 s, 55 °C 45 s, 72 °C 2 min; 35 cycles of 94 °C 45 s, 54 °C 45s, 72 °C 2 min; 72 °C 10 min; 10 °C soak	F8	TTCTTCCACGCCATGGCTGGTCG	O’Donnell et al. (2010)
		G2R	GTCATYTGDGTDGCDGGYTCDCC	O’Donnell et al. (2010)
*RPB2*	95 °C 5 min; 40 cycles of 94 °C 30 s, 51 °C 90 s, 68 °C 2 min; 68 °C 5 min; 10 °C soak	5F2	GGGGWGAYCAGAAGAAGGC	Reeb et al. (2004)
		7Cr	CCCATRGCTTGYTTRCCCAT	Liu et al. (1999)
	95 °C 5 min; 40 cycles of 94 °C 30 s, 51 °C 90 s, 68 °C 2 min; 68 °C 5 min; 10 °C soak	7Cf	ATGGGYAARCAAGCYATGGG	Liu et al. (1999)
		11ar	GCRTGGATCTTRTCRTCSACC	Liu et al. (1999)

* R = A or G; S = C or G; W = A or T; Y = C or T.

### ﻿Phylogenetic analyses

Initial identifications of all *Fusarium* strains relied on BLAST search comparisons against the *Fusarium*-MLST database (https://fusarium.mycobank.org). These results were then used to produce a reference dataset for the FIESC using previously deposited sequences obtained from the NCBI nucleotide database, largely based on O’Donnell et al. (2009) and Xia et al. (2019) (Suppl. material 5). Several phylogenetic trees were computed. The first included selected reference and newly generated sequences, using *TEF*, *CaM* and *RPB2*. Multi- and single-gene trees were calculated from these datasets, with final identifications being based on these trees. We also computed a phylogeny using a more comprehensive dataset based on *TEF*. This phylogeny included all strains obtained from our study, as well as those strains from Avila et al. (2019), Botha et al. (2014), Crous et al. (2021), Lombard et al. (2022), O’Donnell et al. (2009), O’Donnell et al. (2012), Tan and Shivas (2023), Tan and Shivas (2024) and Xia et al. (2019) that contained highly similar identities to ours. The datasets were aligned using MAFFT v. 7.427 (Katoh and Standley 2013) with the G-INS-I option selected in Geneious Prime. For the concatenated dataset, each gene region was treated as a separate partition. Maximum Likelihood (ML) trees were calculated in IQ-TREE v. 2.1.2 (Nguyen et al. 2015) with the General Time Reversible nucleotide substitution model with gamma distribution with invariant sites (GTR+G+I) applied to each partition. Support of nodes was calculated with a standard nonparametric bootstrap analysis with 1,000 replicates (Felsenstein 1985). The resulting trees were visualised using Figtree v. 1.4.4 and edited in Affinity Designer v. 1.7.3 (Serif (Europe) Ltd, Nottingham, UK).

### ﻿Morphological characterisation

*Fusarium* strains were characterised based on macro- and micromorphological features (Leslie and Summerell 2006; Aoki et al. 2013; Sandoval-Denis et al. 2018; Yilmaz et al. 2021). After a 7 d incubation on synthetic nutrient-poor agar (SNA) (Nirenberg 1976) at 25 °C, agar plugs were removed from the colony edges with a 5 mm diameter cork borer and transferred to PDA, oatmeal agar (OA) and SNA for colony morphology and pigmentation assessment. The plates containing the agar plugs were incubated under different light conditions for 7 d, which included 24 h darkness, 24 h near-ultraviolet (nUV) light and a 12/12 h dark/nUV light cycle. Colony colour and codes used in descriptions followed the Methuen Handbook of Colour (Kornerup and Wanscher 1978). Colony growth rates were evaluated on PDA incubated for 7 d at 10–35 °C with 5 °C intervals in 24 h darkness. Colony measurements were recorded daily in four perpendicular directions. Colony images were captured with a Sony Alpha a7 III camera equipped with a Sony FE 90 mm f/2.8 Macro G OSS lens (Tokyo, Japan). Sporodochial formation was evaluated after a 7 d incubation period at 25 °C under a 12/12 h dark/nUV light cycle on SNA and WA, both supplemented with sterilised pieces of carnation leaves. Micromorphological characteristics were examined with a Zeiss AXIO Imager.A2 compound and AXIO Zoom.V16 microscope equipped with an AxioCaM 512 colour camera driven by Zen Blue 3.2 software (Carl Zeiss CMP, Göttingen, Germany). Conidia and other morphological structures were measured using up to 50 measurements each in NIS-Elements Basic Research software v. 4.30.00 (Nikon Europe B.V.). Photographic plates were prepared using Affinity Designer v. 1.7.3 (Serif (Europe) Ltd, Nottingham, UK).

## ﻿Results

### ﻿Identifications and phylogenetic analyses

**Figure 1.**
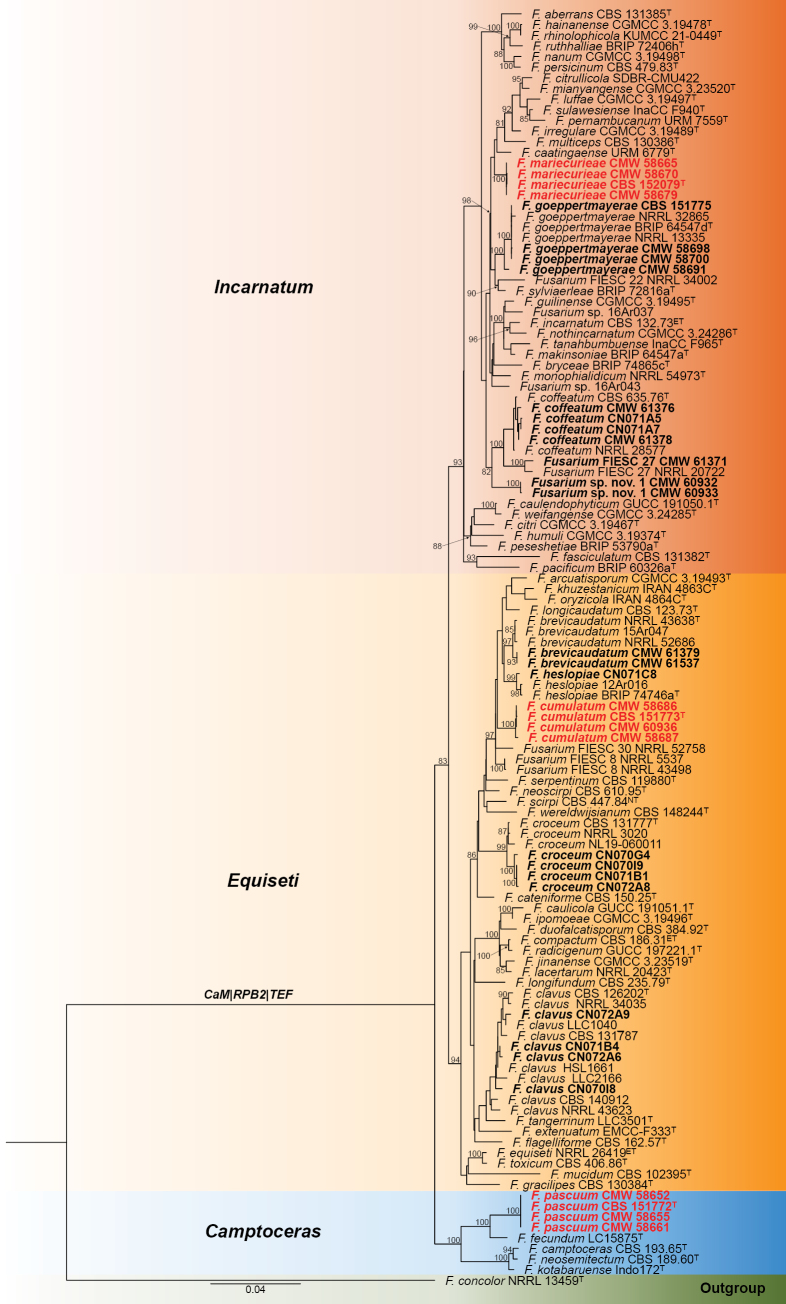
Maximum likelihood phylogenetic tree of the *Fusariumincarnatum-equiseti* species complex based on a concatenated dataset, *CaM, RPB2* and *TEF*. Strains of species isolated from this study are shown in black bold text; strains of new species are indicated in red bold text. The tree was rooted to *Fusariumconcolor*. Branch support in nodes higher than 80% are indicated at relevant branches (^T^ = ex-type, ^ET^ = epitype, ^NT^ = neotype).

Isolations from the 95 mixed pasture grass samples collected in the Eastern Cape resulted in 708 strains isolated, with 55 genera and 133 species identified (Dewing et al. 2025). Of the 207 strains identified as *Fusarium* isolated from 12 of the 14 farms, 155 belonged to the *Fusariumincarnatum*-*equiseti* species complex (FIESC) (Table 1). The aligned concatenated dataset included 120 taxa and was 2,954 bp long (*CaM*: 1–644; *RPB2_1*: 645–1,471; *RPB2_2*: 1,471–2,334; *TEF*: 2,335–2,954). *Fusariumconcolor* (NRRL 13459^T^) was selected as the outgroup. The obtained ML tree resolved strains into three main clades, including the *Incarnatum*-clade, *Equiseti*-clade and *Camptoceras*-clade (O’Donnell et al. 2009; Xia et al. 2019; Han et al. 2023) (Fig. 1). Strains isolated from pastures represented 11 species, with five well-supported clades representing new species. Individual gene phylogenies for *CaM*, *RPB2* and *TEF* were used to assess these clades, applying genealogical concordance phylogenetic species recognition (GCPSR: Taylor et al. (2000)) (Suppl. materials 1–3). In all analyses, strains of the new species showed no discordance, noting that the branches holding *F.mariecurieae* did not have support in the *CaM* and *RPB2* phylogenies.

*Incarnatum*-clade—We identified five species in the *Incarnatum*-clade, including *F.coffeatum* (FIESC 28; n = 7), *F.goeppertmayerae* (n = 14) and the clade we introduce as *F.mariecurieae* (n = 33) below (Fig. 1, Suppl. materials 1–3). Strains CMW 58691 and CMW 60930 showed variation in *TEF* by at least 11 bp but had similar *CaM* and *RPB2* sequences from other *F.goeppertmayerae* strains. *Fusariumgoeppertmayerae* was described based on a single isolate (BRIP 64547d^T^), and capturing this type of infraspecies variation is important to better establish its species boundaries. We provide a description of the species below in the Taxonomy section because Tan and Shivas (2023) did not provide this when naming their species. Two additional new species were isolated, including *Fusarium*FIESC 27 (O’Donnell et al. 2009) (n = 1) and *Fusarium* sp. nov. 1 (n = 2). However, due to the absence of key microscopic characteristics, we opted to not introduce names for these until additional strains are collected.

*Equiseti*-clade—Five of our species were resolved in the *Equiseti*-clade, from which *F.brevicaudatum* (FIESC 6; n = 2), *F.clavus* (FIESC 5; n = 19), *F.croceum* (FIESC 10; n = 55) and *F.heslopiae* (n = 1) are known species, while *F.cumulatum* (n = 4) is described below as a new species (Fig. 1, Suppl. materials 1–3). The *F.clavus* clade contains a lot of variation. In the *TEF* phylogeny, *F.clavus* strains form a unique group with *F.tangerrinum*, its closest relative. However, in *RPB2*, both *F.tangerrinum* and *F.extenuatum* resolve inside the broader *F.clavus* clade. In the *CaM* phylogeny, *F.tangerrinum* also resolves inside *F.clavus*, but *F.extenuatum* is a distant relative. Future work is needed on this group. Our *F.croceum* strains consistently formed a distinct clade closely related to other *F.croceum* strains (including the ex-type CBS 131777^T^), with ours differing by at least 9, 8 and 27 bp for *CaM*, *RPB2* and *TEF*, respectively. Based on these findings, we could introduce a new species for the clade. However, at present we propose that our strains represent new genotypes of *F.croceum* with additional strains that will be needed in the future. Finally, we identify strain CN071C8 as *F.heslopiae*, a species originally introduced based solely on a *TEF* sequence, with no morphological description provided (Tan and Shivas 2024).

*Camptoceras*-clade—We identified a new species in the *Camptoceras*-clade, which we introduce below as *F.pascuum* (n = 17) (Fig. 1, Suppl. materials 1–3). The new species is a close relative of *F.fecundum*.

### ﻿Taxonomy

#### ﻿ Fusarium cumulatum

Taxon classificationFungiHypocrealesNectriaceae

Dewing, Visagie & Yilmaz sp. nov.

124430B7-210F-57BE-8B3F-FE27B80C84AA

MycoBank No: 855721

Fig. 2

##### Etymology.

Latin, *cumulatum*, meaning to accumulate or heap up, named for its abundant chlamydospore formation.

##### Type.

South Africa • Eastern Cape, from mixed pasture samples, May 2020, collected by A. Davis (***holotype***: PRU(M) 4601, dried specimen in a metabolically inactive state); (***ex-type strain***: CBS 151773 = CMW 58688 = CN104D3).

**Figure 2.**
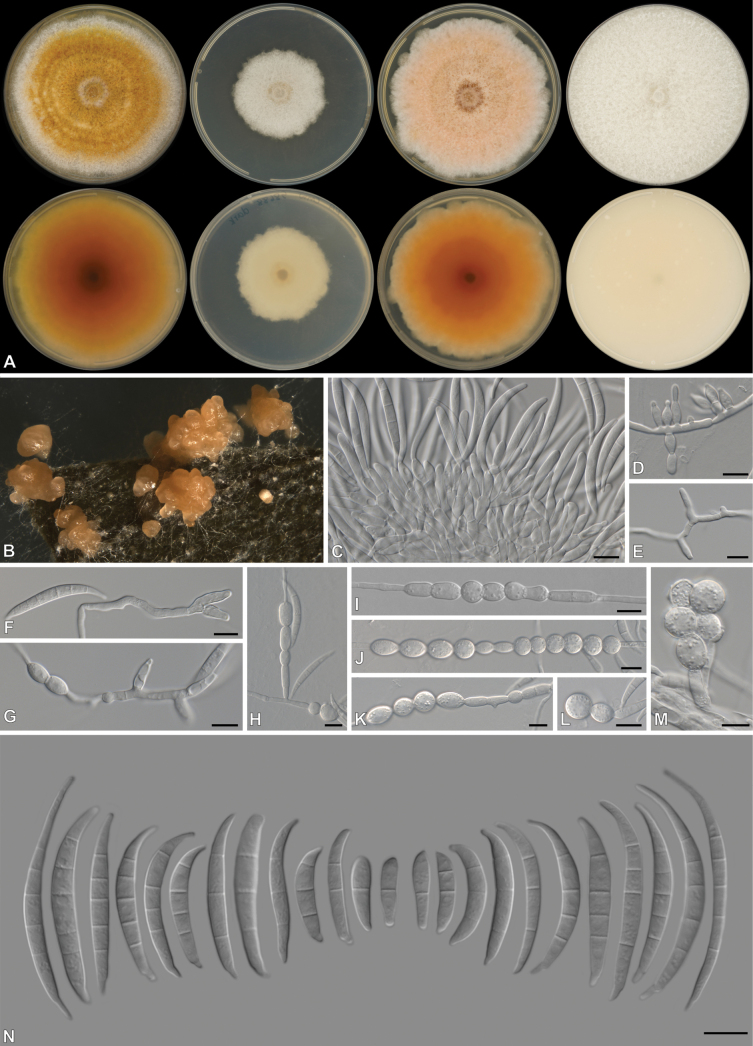
*Fusariumcumulatum* (CBS 151773, ex-type culture) **A** colonies front (top row) and reverse (bottom row) on PDA after 7 d at 25 °C light, dark and nUV and OA after 7 d at 25 °C dark (from left to right), respectively **B** sporodochial formation on the surface of carnation leaves **C, D** sporodochial Sporodochial conidiophores and phialides **E–G** aerial Sporodochial conidiophores and phialides **H–M** intercalary and terminal chlamydospores **N** sporodochial conidia. Scale bars: 10 μm.

##### Description.

***Conidiophores*** borne on aerial mycelium scarce, 13–71 μm tall, unbranched, bearing terminal phialides, often reduced to single phialides; ***aerial phialides*** scarce, monophialidic, subulate to subcylindrical, proliferating percurrently, smooth- and thin-walled, 2.5–20 × 2–4 μm, with inconspicuous thickening; ***aerial conidia*** absent. ***Sporodochia*** orange, present on the surface of carnation leaves and on agar. ***Sporodochial conidiophores*** densely and irregularly branched, bearing apical whorls of 2–5 phialides; ***sporodochial phialides*** monophialidic, subulate to subcylindrical, 7–16.5 × 2–4 μm, smooth, thin-walled, with inconspicuous periclinal thickening; ***sporodochial conidia*** falcate, sometimes becoming sinuate, slender, curved dorsiventrally, tapering towards both ends, with an elongated or whip-like curved apical cell and a barely notched to prominently extended basal cell, 1–5-septate, hyaline, smooth- and thin-walled; 1-septate conidia 16 × 4 μm (n = 1); 2-septate conidia 18–30 × 3–4 μm (av. 25.2 × 3.6 μm) (n = 3), 3-septate conidia 23–42 × 2.5–4 μm (av. 25.2 × 3.5 μm) (n = 15), 4-septate conidia 25.5–54.5 × 2.5–4 μm (av. 43.0 × 3.4 μm) (n = 14), 5-septate conidia 38–57 × 3–4.5 μm (av. 49.1 × 3.8 μm) (n = 17). ***Chlamydospores*** abundant, globose to subglobose, subhyaline, smooth- to slightly rough-walled, terminal or intercalary, solitary or in pairs forming chains, 8–19 μm diam.

##### Culture characteristics.

Colonies on PDA incubated at 25 °C in the dark with an average radial growth rate of 2–8 mm/d, reaching 44–46 mm diam at 25 °C; surface white, flat, felty to velvety, radiate, with abundant aerial mycelium, margin irregular. Additional colony diam (after 7 d, in mm): PDA 10 °C 13–15; PDA at 15 °C 22–26; PDA at 20 °C 27–32; PDA at 30 °C 64–75; PDA at 35 °C 0–2. Odour absent. Reverse yellowish white (2A2). Diffusible pigments absent. On OA in the dark, occupying an entire 90 mm Petri dish in 7 d; surface white to pale yellow, flat, felty to velvety, radiate, with abundant aerial mycelium, margin irregular, filiform. Reverse yellowish white (4A2). Diffusible pigments absent. On SNA with sparse aerial mycelium, sporulation moderate on the surface of the medium.

##### Additional materials examined.

South Africa • Eastern Cape, from mixed pasture samples, May 2020, collected by A. Davis, isolated by C. Dewing, Humansdorp area: CMW 58686 = CN071B9, CMW 58687 = CN071E5, close to Villa Fonte: CMW-IA 002138 = CMW 60936 = CN071G4.

##### Notes.

*Fusariumcumulatum* belongs to the *Equiseti*-clade and is closely related to *F.arcuatisporum* (FIESC 7) (Wang et al. 2019), *F.brevicaudatum* (FIESC 6) (Xia et al. 2019), *F.heslopiae* (Tan and Shivas 2024), *F.longicaudatum* (Xia et al. 2019), *F.khuzestanicum* and *F.oryzicola* (Afzalinia et al. 2024). No aerial phialides or conidia were observed for the closely related species (Wang et al. 2019; Xia et al. 2019; Afzalinia et al. 2024) compared to the few scarce monophialides we recorded for *F.cumulatum*. Sporodochia and chlamydospores were present in *F.cumulatum* and its closely related species, whereas *F.oryzicola* lacked chlamydospore formation (Wang et al. 2019; Xia et al. 2019; Afzalinia et al. 2024). Sporodochial conidia of *F.cumulatum* (1–5-septate; 16–57 × 2.5–4 μm) are similar in length to *F.arcuatisporum* (5-septate; 29–49.5 × 4–6 μm) (Wang et al. 2019) and *F.brevicaudatum* (1–5-septate; 8–64 × 3–5 μm) (Xia et al. 2019), while generally being shorter than those observed in *F.khuzestanicum* (4–7(–9)-septate; 48.5–82 × 2.7–4.3 μm) (Afzalinia et al. 2024), *F.longicaudatum* ((3–)5–6(–7)-septate; 45–81 × 4–5 μm) (Xia et al. 2019) and *F.oryzicola* (4–7-septate; 33.5–77.9 × 3–4 μm) (Afzalinia et al. 2024). Colony colour on PDA differs between *F.cumulatum* and closely related species (Wang et al. 2019; Xia et al. 2019; Afzalinia et al. 2024) as other species show more colour across the surface and reverse compared to the white surface and yellowish white (2A2) reverse of *F.cumulatum*, whereas the colony colour of *F.khuzestanicum* and *F.oryzicola* is white to pale grey. The growth rate after 7 d on PDA for *F.cumulatum* (44–46 mm) is slower than that of *F.arcuatisporum* (48–53 mm) (Wang et al. 2019), *F.brevicaudatum* (50–58 mm) (Xia et al. 2019) and *F.longicaudatum* (full 90 mm plate) (Xia et al. 2019). The growth rate for *F.khuzestanicum* (74–76 mm) (Afzalinia et al. 2024) and *F.oryzicola* (74 mm) (Afzalinia et al. 2024) was measured after 5 d on PDA but appears to be faster than that of *F.cumulatum*. No morphological data is currently available for *F.heslopiae* to compare with. Pairwise comparisons revealed that *F.cumulatum* differs from other species by at least 3, 6 and 16 bp for *CaM*, *RPB2* and *TEF*, respectively.

#### ﻿ Fusarium goeppertmayerae

Taxon classificationFungiHypocrealesNectriaceae

Y.P. Tan & R.G. Shivas, Index of Australian Fungi 5: 7. 2023.

3550AD2E-C689-5555-A00D-206A1BFBFB74

MycoBank No: 900363

Fig. 3

##### Type.

Australia • Queensland, Bongeen, from the peduncle of *Zeamays* (Poaceae), 25 Feb. 2016, B. Thrift (***holotype***: BRIP 64547d, ***ex-type***: CBS 150772).

**Figure 3.**
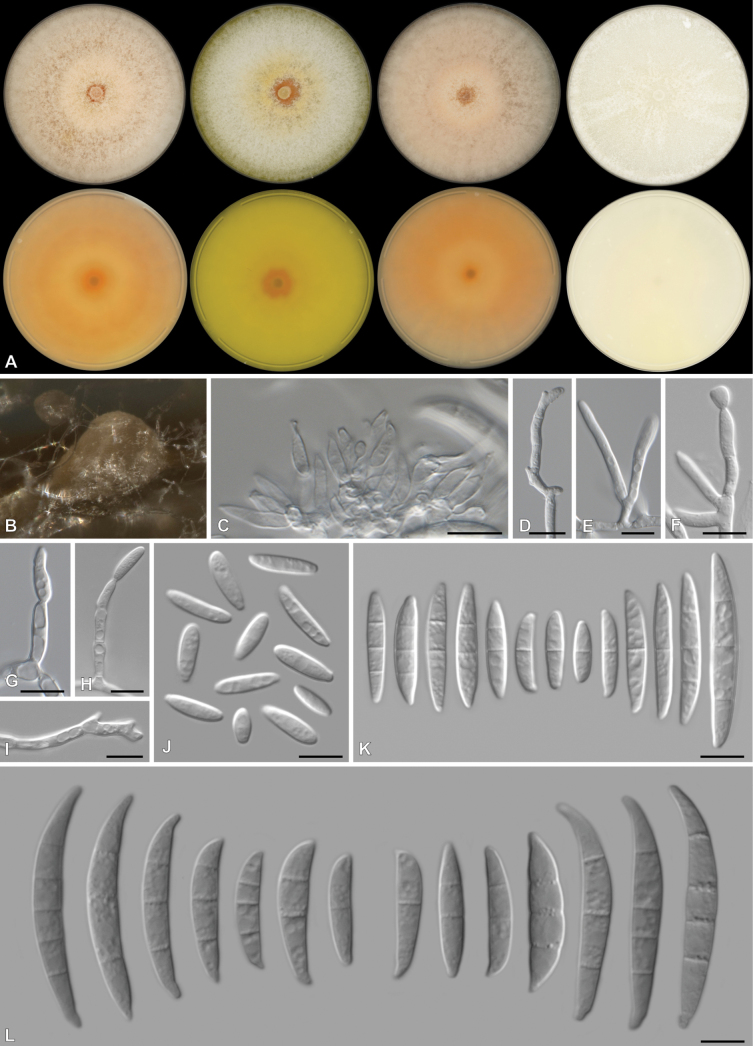
*Fusariumgoeppertmayerae* (CBS 151775) **A** colonies front (top row) and reverse (bottom row) on PDA after 7 d at 25 °C light, dark and nUV and OA after 7 d at 25 °C dark (from left to right), respectively **B** sporodochial formation on the surface of carnation leaves **C** sporodochial Sporodochial conidiophores **D–I** aerial mono- and polyphialides **J–K** aerial conidia **L** sporodochial conidia. Scale bars: 10 μm.

##### Description.

***Conidiophores*** borne on aerial mycelium, 8.5–98 um tall, unbranched, sympodial, bearing terminal or lateral phialides, often reduced to single phialides; ***aerial phialides*** mono- and polyphialidic, subulate to subcylindrical, proliferating percurrently, smooth- and thin-walled, 4–22 × 1.5–5 μm, with inconspicuous thickening; ***aerial conidia*** mostly fusiform, slender, curved dorsiventrally, no apparent tapering observed at ends, blunt to conical and straight to slightly curved apical cell and a blunt to papillate basal cell, 0–3-septate, 0-septate conidia: 7–22 × 2–5 μm (av. 15.0 × 3.4 μm) (n = 9); 1-septate conidia: 12–19 × 2.5–4 μm (av. 15.6 × 3.4 μm) (n = 13); 2-septate conidia: 16–20 × 3.5–4 μm (av. 18.2 × 3.8 μm) (n = 2); 3-septate conidia: 21–31 × 3.5–4 μm (av. 23.9 × 3.9 μm) (n = 6). ***Sporodochia*** pale yellow to white, formed between aerial mycelia around the carnation leaves. ***Sporodochial conidiophores*** densely and irregularly branched, bearing apical whorls of 2–3 phialides; ***sporodochial phialides*** monophialidic, subulate to subcylindrical, 6–12 × 1.5–4 μm, smooth, thin-walled, with inconspicuous periclinal thickening; ***sporodochial conidia*** falcate, curved dorsiventrally, tapering towards both ends, with a slightly curved apical cell and a blunt to foot-like basal cell, (1–)3–5-septate, hyaline, smooth- and thin-walled; 1-septate conidia: 12–17 × 3 μm (av. 14.4 × 3.2 μm) (n = 2); 3-septate conidia: 19–36 × 3 × 4 μm (av. 30.0 × 3.8 μm) (n = 23); 4-septate conidia: 30.5–36 × 4–5 μm (av. 33.2 × 4.3 μm) (n = 4); 5-septate conidia: 30 × 5 μm (n = 1). ***Chlamydospores*** not observed.

##### Culture characteristics.

Colonies on PDA incubated at 25 °C in the dark with an average radial growth rate of 1–15 mm/d and occupying an entire 90 mm Petri dish in 7 d; surface white, radiate, aerial mycelium felty to velvety, margin irregular, filiform. Additional colony diam (after 7 d, in mm): PDA at 10 °C 14–19; PDA at 15 °C 37–43; PDA at 20 °C 63–70; PDA at 30 °C 40–75; PDA at 35 °C 0–2. Odour absent. Reverse pale yellow. Diffusible pigments absent. On OA in the dark, occupying an entire 90 mm Petri dish in 7 d; surface white, flat, slightly felty to velvety, aerial mycelium scant, margin irregular, filiform. Reverse pale luteous, without diffusible pigments. On SNA with sparse aerial mycelium, sporulation moderate on the surface of the medium.

##### Materials examined.

South Africa • Eastern Cape, from mixed pasture samples, May 2020, collected by A. Davis, isolated by C. Dewing, close to Gamtoos River Mouth: CBS 151775 = CMW 58689 = CN040I5, Outside Humansdorp, close to Clarkson: CMW 58690 = CN070F3, CMW 58696 = CN071I8, CMW-IA 003340 = CMW 61384 = CN071H2, CMW 58693 = CN071H8, Humansdorp area: CMW 58691 = CN070G8, CMW 58692 = CN070G9, CMW-IA 002132 = CMW 60930 = CN070H9, CMW 58697 = CN104D4, CMW 58698 = CN106F2, CMW 58699 = CN106F3, CMW 58700 = CN106F4, close to Tsitsikamma on Sea: CMW 58694 = CN071I6, CMW 58695 = CN071I7, CMW 58696 = CN071I8.

##### Notes.

*Fusariumgoeppertmayerae* belongs to the *Incarnatum*-clade and is closely related to the undescribed *Fusarium*FIESC 22 isolated from the human sinus cavity (O’Donnell et al. 2009) and *F.sylviaearleae* isolated from a leaf lesion of *Sporobolusnatalensis* (Poaceae) (Tan and Shivas 2023). No morphological data are available for *Fusarium*FIESC 22 or *F.sylviaearleae*. Furthermore, we demonstrate that strains NRRL 32865 and NRRL 13335, previously considered to belong to *F.guilinense*, belong to *F.goeppertmayerae*, with *F.guilinense* (LC12160^T^) a distant relative.

#### ﻿ Fusarium mariecurieae

Taxon classificationFungiHypocrealesNectriaceae

Dewing, Visagie & Yilmaz sp. nov.

4902A011-30F7-51AB-99D4-3C5556E77893

MycoBank No: 855722

Fig. 4

##### Etymology.

Latin, *mariecurieae*, named after Maria Salomea Skłodowska-Curie (known simply as Marie Curie) (1867–1934), who was a renowned physicist and chemist known for her pioneering research on radioactivity. We also chose this name, as this study was supported by the Marie Skłodowska‐Curie Actions (MSCA) grant (number 101008129), project acronym “Mycobiomics”.

**Figure 4.**
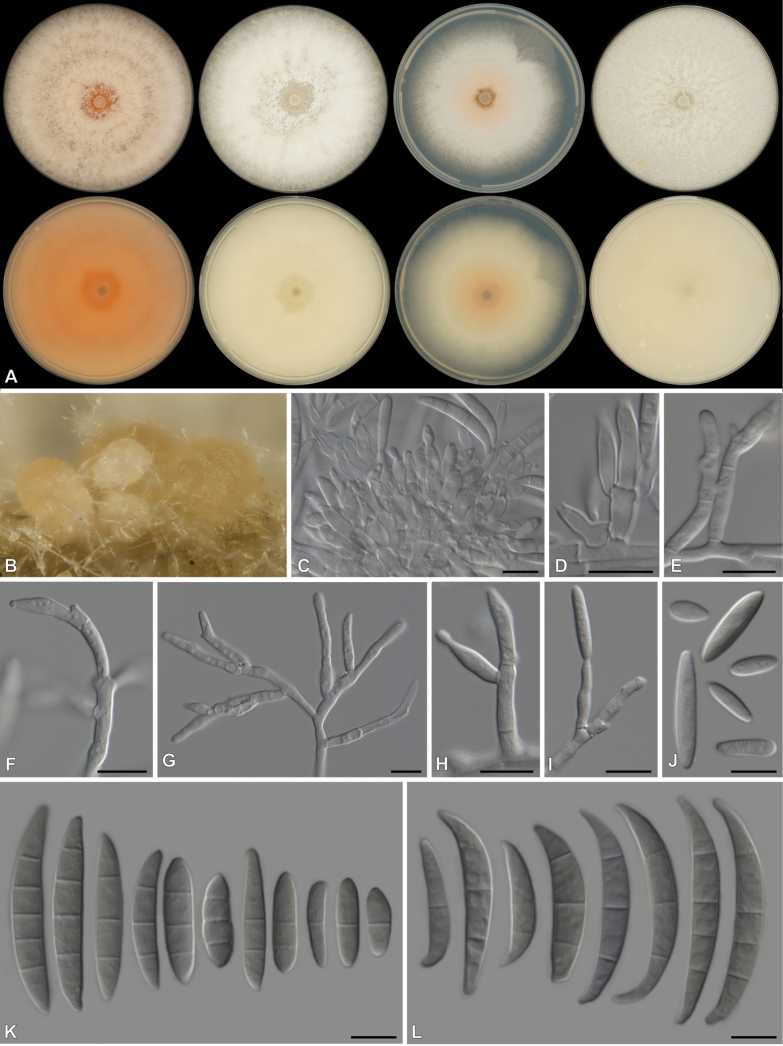
*Fusariummariecurieae* (CBS 152079, ex-type culture) **A** colonies front (top row) and reverse (bottom row) on PDA after 7 d at 25 °C light, dark and nUV and OA after 7 d at 25 °C dark (from left to right), respectively **B** sporodochial formation on the surface of carnation leaves **C, D** sporodochial Sporodochial conidiophores and phialides **E–G** aerial Sporodochial conidiophores **H–I** mono- and polyphialides **J–K** aerial conidia **L** sporodochial conidia. Scale bars: 10 μm.

##### Type.

South Africa • Eastern Cape, from mixed pasture samples, May 2020, collected by A. Davis (***holotype***: PRU(M) 4611, dried specimen in a metabolically inactive state; ***ex-type strain***: CBS 152079 = CMW 58673 = CN072A3).

##### Description.

***Conidiophores*** borne on aerial mycelium, 13–106 μm tall, unbranched, sympodial or irregularly branched, bearing terminal or lateral phialides, often reduced to single phialides; ***aerial phialides*** mono- and polyphialidic, subulate to subcylindrical, proliferating percurrently, smooth- and thin-walled, 3.5–28.5 × 1.5–4 μm, with inconspicuous thickening; ***aerial conidia*** ellipsoidal, fusiform, slightly allantoid to falcate, slender, curved dorsiventrally and more pronounced on the apical half, tapering towards both ends, with a blunt to conical and straight to slightly curved apical cell and a blunt to papillate basal cell, 0–3(–5)-septate; 0-septate conidia: 8–11 × 2.5–3 μm (av. 9.6 × 2.6 μm) (n = 2); 1-septate conidia: 11–20 × 3–4 μm (av. 15.6 × 3.3 μm) (n = 11); 2-septate conidia: 15–23 × 3–4 μm (av. 18.7 × 3.6 μm) (n = 6); 3-septate conidia: 18.5–30.5 × 3–5 μm (av. 23.2 × 3.8 μm) (n = 26); 5-septate conidia: 33 × 5 μm (n = 1). ***Sporodochia*** peach to pale straw, formed abundantly on carnation leaves. ***Sporodochial conidiophores*** densely and irregularly branched, bearing apical whorls of 2–3 phialides; ***sporodochial phialides*** monophialidic, subulate to subcylindrical, 6–22 × 2–4 μm, smooth, thin-walled, with inconspicuous periclinal thickening; ***sporodochial conidia*** falcate, curved dorsiventrally, tapering towards both ends, with a slightly curved apical cell and a blunt to foot-like basal cell, (1–)3–5-septate, hyaline, smooth- and thin-walled; 1-septate conidia: 12–17 × 3 μm (av. 14.4 × 3.2 μm) (n = 2); 3-septate conidia: 19–36 × 3–4 μm (av. 30.0 × 3.8 μm) (n = 23); 4-septate conidia: 31–36 × 4–5 μm (av. 33.2 × 4.3 μm) (n = 4); 5-septate conidia: 30 × 5 μm (n = 1). ***Chlamydospores*** not observed.

##### Culture characteristics.

Colonies on PDA incubated at 25 °C in the dark with an average radial growth rate of 5–9 mm/d, occupying an entire 90 mm Petri dish in 7 d; surface white, flat, felty to velvety around the centre, floccose towards the margins, radiate, with abundant aerial mycelium, margin irregular, filiform. Additional colony diam (after 7 d): PDA 10 °C 12–17; PDA at 15 °C 29–40; PDA at 20 °C 48–70; PDA at 30 °C 68–76; PDA at 35 °C 4–6. Odour absent. Reverse yellowish white (3A2). Diffusible pigments absent. On OA in the dark, occupying an entire 90 mm Petri dish in 7 d; surface white, floccose around the centre, flat, felty to velvety towards the margin, radiate, with abundant aerial mycelium, margin irregular, filiform. Reverse yellowish white (2A2). Diffusible pigments absent. On SNA with sparse aerial mycelium, sporulation moderate on the surface of the medium.

##### Additional materials examined.

South Africa • Eastern Cape, from mixed pasture samples, May 2020, collected by A. Davis, isolated by C. Dewing, Humansdorp area: CMW 58664 = CN070E5, CMW-IA 002131 = CMW 60929 = CN070F5, CMW-IA 003328 = CMW 61372 = CN070G5, CMW 58666 = CN070H6, CBS 151774 = CMW 58667 = CN070I7, CMW 58668 = CN071B2, CMW 58669 = CN071C1, CMW 58670 = CN071E4, CMW-IA 002136 = CMW 60934 = CN071F2, CMW-IA 002137 = CMW 60935 = CN071F3, CMW 58671 = CN071F4, CMW 58676 = CN072E2, CMW 58677 = CN104C2, CMW 58678 = CN104E1, CMW 58679 = CN106F1, CMW 58680 = CN106F8, CMW-IA 003763 = CMW 61535 = CN106F9, CMW 58681 = CN106G1, CMW 58682 = CN106G2, CMW 58683 = CN106G3, CMW 58684 = CN106G4, CN106G5, CMW-IA 003764 = CMW 61536 = CN110D9, CMW 58685 = CN110E2, CN115C6, CN115C9, CN115D4, CN115E8, CMW 61371 = CN070G1, Outside Humansdorp, close to Clarkson: CMW 58665 = CN070G2, CMW 58672 = CN071H1, CMW 58674 = CN072B2, close to Villa Fonte: CMW 58675 = CN072B6.

##### Notes.

*Fusariummariecurieae* belongs to the *Incarnatum*-clade and is most similar to an unsupported clade containing the following species: *F.caatingaense* (FIESC 20) (Santos et al. 2019), *F.citrullicola* (nom. inval.) (Khuna et al. 2022), *F.irregulare* (FIESC 15) (Wang et al. 2019), *F.luffae* (FIESC 18) (Wang et al. 2019), *F.mianyagense* (Han et al. 2023), *F.multiceps* (FIESC 19) (Xia et al. 2019), *F.pernambucanum* (FIESC 17) (Santos et al. 2019) and *F.sulawesiense* (FIESC 16) (Maryani et al. 2019). *Fusariummariecurieae* produces both aerial mono- and polyphialides compared to *F.irregulare* that only produces monophialides (Wang et al. 2019), *F.luffae* that produces only polyphialides (Wang et al. 2019) and *F.mianyagense* that lacks aerial phialides (Han et al. 2023). Aerial conidia from *F.mariecurieae* (0–3(–5)-septate; 8–30.5 × 3–5 μm) are smaller than that of *F.irregulare* (mostly 3-septate; 16–38.5 × 3–5 μm) (Wang et al. 2019), *F.luffae* (3–5)-septate; 26.5–46 × 4–5 μm) (Wang et al. 2019), *F.multiceps* (1–)3–4(–5)-septate; 16–37 × 3–4 μm) (Xia et al. 2019), *F.pernambucanum* (1–7)-septate; 7–57 × 2.5–5 μm) (Santos et al. 2019) and *F.sulawesiense* (3–5(–9)-septate; 20.5–67 × 3.5–6 μm) (Maryani et al. 2019). Aerial conidia from *F.caatingaense* (0–6-septate; 6–45 × 2.5–5 μm) (Santos et al. 2019) and *F.citrullicola* (1–5-septate; 8–39 × 2–4.9 μm) (Khuna et al. 2022) were, at their largest, bigger than those of *F.mariecurieae*, while aerial conidia were absent from *F.mianyagense* (Han et al. 2023). Sporodochia were absent from *F.citrullicola*, *F.irregulare* and *F.luffae* (Wang et al. 2019), while chlamydospores were absent from *F.irregulare*, *F.luffae*, *F.mianyagense*, *F.multiceps* and *F.sulawesiense* (Maryani et al. 2019; Wang et al. 2019; Xia et al. 2019; Han et al. 2023). Sporodochial conidia from *F.mariecurieae* (1–3(–5)-septate; 12–36 × 3–5 μm) were smaller than that of *F.caatingaense* (1–5-septate; 15–50 × 2–4.5 μm) (Santos et al. 2019), *F.mianyagense* (3(–5)-septate; 24.5–36.6 × 2.5–4.9 μm) (Han et al. 2023), *F.multiceps* ((1–)2–5-septate; 16–46 × 3–4 μm) (Xia et al. 2019) and *F.sulawesiense* ((3–)5(–6)-septate; 29.5–43.5 × 4–5.5 μm) (Maryani et al. 2019). Colony colour on PDA differs between *F.mariecurieae* and closely related species (Maryani et al. 2019; Santos et al. 2019; Wang et al. 2019; Xia et al. 2019; Han et al. 2023) as most other species show more colour across the surface and reverse compared to the white surface and yellowish white (3A2) reverse of *F.mariecurieae*. The growth rate after 7 d on PDA for *F.mariecurieae* is faster (>90 mm plate) than that of *F.citrullicola* (68–74.5 mm) (Khuna et al. 2022), *F.irregulare* (53–59 mm) (Wang et al. 2019), *F.luffae* (53–57 mm) (Wang et al. 2019) and *F.mianyagense* (74–80 mm) (Han et al. 2023). The growth of *F.multiceps* (>90 mm plate) (Xia et al. 2019) is similar to that of *F.mariecurieae*, while the growth rate in terms of diameter was not reported for *F.caatingaense*, *F.pernambucanum* and *F.sulawesiense* (Maryani et al. 2019; Santos et al. 2019). Pairwise comparisons revealed that *F.mariecurieae* differs from other species by at least 1, 4 and 12 bp for *CaM*, *RPB2* and *TEF*, respectively.

#### ﻿ Fusarium pascuum

Taxon classificationFungiHypocrealesNectriaceae

Dewing, Visagie & Yilmaz sp. nov.

16A91355-E273-52B7-A936-E46F714C5C80

MycoBank No: 855720

Fig. 5

##### Etymology.

Latin, *pascuum*, meaning pasture, referring to the species isolated from grass pastures.

##### Type.

South Africa • Eastern Cape, from mixed pasture samples, May 2020, collected by A. Davis (***holotype***: PRU(M) 4600, dried specimen in a metabolically inactive state; ***ex-type strain***: CBS 151772 = CMW 58653 = CN159G4 = CN071C4).

**Figure 5.**
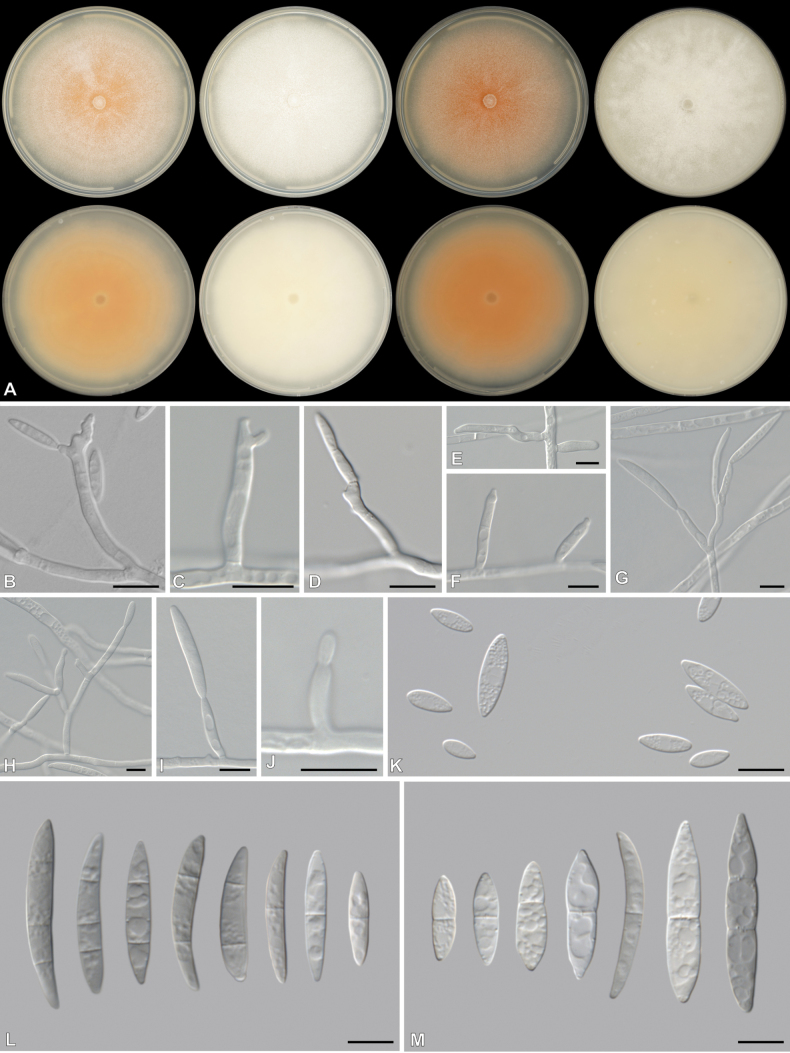
*Fusariumpascuum* (CBS 151772, ex-type culture) **A** colonies front (top row) and reverse (bottom row) on PDA after 7 d at 25 °C light, dark and nUV and OA after 7 d at 25 °C dark (from left to right), respectively **B–J** aerial Sporodochial conidiophores and phialides **K** aerial microconidia **L, M** aerial macroconidia. Scale bars: 10 μm.

##### Description.

***Conidiophores*** borne on aerial mycelium, 15.5–101 μm tall, unbranched, sympodial or irregularly branched, bearing terminal or lateral phialides, often reduced to single phialides; ***aerial phialides*** mono- and polyphialidic, subulate to subcylindrical, proliferating percurrently, smooth- and thin-walled, 4–43 × 1–4.5 μm, with inconspicuous periclinal thickening; ***aerial conidia*** fusiform, falcate, slender, curved dorsiventrally and more pronounced on the apical half, tapering towards both ends, with a blunt to conical and straight to slightly curved apical cell and a blunt to papillate basal cell, 0–3-septate conidia; 0-septate conidia: 7–17 × 2–5 μm (av. 11.7 × 3.2 μm) (n = 34); 1-septate conidia: 12–26 × 3–6 μm (av. 19.2 × 3.8 μm) (n = 14); 2-septate conidia: 23–32 × 4–6 μm (av. 26.9 × 4.5 μm) (n = 7); 3-septate conidia: 27–32 × 3–5 μm (av. 29.5 × 4.4 μm) (n = 2). ***Sporodochia*** and ***chlamydospores*** not observed.

##### Culture characteristics.

Colonies on PDA incubated at 25 °C in the dark with an average radial growth rate of 3–10 mm/d, reaching 80 mm diam at 25 °C; surface white, flat, felty to velvety, radiate, with abundant aerial mycelium, margin irregular, filiform. Additional colony diam (after 7 d, in mm): PDA at 10 °C 13–15; PDA at 15 °C 36–42; PDA at 20 °C 63–65; PDA at 30 °C 34–39; PDA at 35 °C no growth. Odour absent. Reverse yellowish white (3A2). Diffusible pigments absent. On OA in the dark, occupying an entire 90 mm Petri dish in 7 d; surface white, flat, felty to velvety, radiate, with abundant aerial mycelium, margin irregular, filiform. Reverse yellowish white (3A2). Diffusible pigments absent. On SNA with sparse aerial mycelium, sporulation moderate on the surface of the medium.

##### Additional materials examined.

South Africa • Eastern Cape, from mixed pasture samples, May 2020, collected by A. Davis, isolated by C. Dewing, Humansdorp area: CMW-IA 003320 = CMW 61364 = CN056A8, CMW 58649 = CN070E7, CMW 58650 = CN070F7, CMW-IA 002133 = CMW 60931 = CN070I3, CMW 58651 = CN070I4, CMW 58652 = CN071B8, CMW 58654 = CN071D3, CMW 58655 = CN071E9, CMW 58662 = CN104D6, CMW 58663 = CN104D7, close to Kou-Kamma: CMW 58656 = CN071F9, Outside Humansdorp, close to Clarkson: CMW 58657 = CN071G8, CMW 58660 = CN071I9, CMW 58661 = CN072A1, close to Tsitsikamma on Sea: CMW 58658 = CN071I3, CMW 58659 = CN071I5.

##### Notes.

*Fusariumpascuum* belongs to the *Camptoceras*-clade (as introduced by Han et al. (2023)) and is closely related to *F.fecundum*. Both *F.pascuum* and *F.fecundum* produce aerial mono- and polyphialides. Aerial conidia from *F.pascuum* (0–3-septate; 7–32 × 2–6 μm) are comparably smaller than those of *F.fecundum* ((1–)2–4(–6)-septate; 3.6–35.8 × 3.6–6.8 μm) (Han et al. 2023). Sporodochia and chlamydospores are absent in both *F.pascuum* and *F.fecundum*. Colony colour on PDA differs between *F.pascuum* and *F.fecundum*, where the former is completely white across the surface and the latter is greyish yellow in the centre, while the reverse of *F.pascuum* is yellowish white and *F.fecundum* is just white (Han et al. 2023). The growth rate after 7 d on PDA in *F.pascuum* (reaching 80 mm) is slightly slower than that of *F.fecundum* (84–90 mm) (Han et al. 2023). Pairwise comparisons revealed that *F.pascuum* differs from other species by at least 8, 8 and 23 bp for *CaM*, *RPB2* and *TEF*, respectively.

## ﻿Discussion

In a 2020 survey exploring fungal diversity in dairy pastures, 95 mixed pasture samples were collected across 14 dairy farms in the Eastern Cape of South Africa. A total of 155 *Fusarium* strains, belonging to the *Fusariumincarnatum-equiseti* species complex (FIESC), were isolated from 12/14 dairy farms. Strains were analysed using a multigene phylogenetic approach, leading to the identification of 11 species, including five that are new. Of these, we opted to formally describe and name *F.cumulatum*, *F.mariecurieae* and *F.pascuum*. *Fusariumcroceum* (n = 55) and *F.mariecurieae* (n = 33) were the most commonly isolated species, followed by *F.clavus* (n = 19), *F.pascuum* (n = 17), *F.goeppertmayerae* (n = 14), *F.coffeatum* (n = 7), *F.cumulatum* (n = 4), *F.brevicaudatum* (n = 2), *Fusarium* sp. nov. 1 (n = 2), *F.heslopiae* (n = 1) and *Fusarium*FIESC 27 (n = 1). Due to a lack of morphological character development in our strains, two of the new species were not described (e.g., *Fusarium*FIESC 27, *Fusarium* sp. nov. 1). In the future, it will be important to obtain additional isolates of the species and name them. Several recently FIESC-introduced species did not include morphological descriptions. This includes *F.goeppertmayerae* that was introduced based on sequence differences of a *TEF* sequence in a single isolate (Tan and Shivas 2023). We found several new isolates of this species in pasture samples, and here we provided a morphological description for the species and capture infraspecies variation in its DNA sequences.

*Fusarium* species are well-known for their frequent association with Poaceae (grasses), but of the 11 species identified, only five had previously been reported from this plant family. *Fusariumclavus* was reported from *Phalarisminor* (little seed canary grass), *Leucopoasclerophylla*, *Secalemontanum* (wild perennial rye) and *Triticum* (wheat) from Iran (Xia et al. 2019). *Fusariumcoffeatum* and *F.heslopiae* were reported from *Cynodonnlemfuensis* (African Bermuda-grass) and *Sporoboluscreber* (Western Rat-Tail grass), respectively (Lombard et al. 2019; Tan and Shivas 2024), while *F.croceum* has been isolated from wheat (*Triticum* sp.) from Iran (Xia et al. 2019). *Fusariumgoeppertmayerae* has not previously been reported from grass species but was reported from maize peduncles from Australia (Tan and Shivas 2023). Given the diverse impacts of FIESC species, especially with regard to their ability to cause plant and animal diseases and produce mycotoxins (Kosiak et al. 2005; Desjardins 2006; O’Donnell et al. 2013; Villani et al. 2016; Munkvold 2017; O’Donnell et al. 2018; Gallo et al. 2022), it is crucial to investigate the potential implications that species present in our dairy pastures could have for animal health. This is especially relevant considering the growing evidence of *Fusarium* species contributing to toxic effects in livestock grazing on certain grasses (Kellerman et al. 2005; Bourke 2007).

Species previously implicated in kikuyu poisoning were identified in our study. Previous studies have identified *Fusarium* species as potential causal agents of kikuyu poisoning, a condition characterised by toxic effects in livestock, like cattle, that consume kikuyu grass (*Pennisetumclandestinum*) (Kellerman et al. 2005; Bourke 2007). Reports of kikuyu poisoning are sporadic in South Africa and Australia and pose significant economic concerns in dairy farming due to high cattle mortality rates (Kellerman et al. 2005; Bourke 2007). While the exact cause of kikuyu poisoning remains uncertain, Ryley et al. (2007) hypothesised that mycotoxins like wortmannin and butenolide produced by species like *F.torulosum* (*Fusariumtricinctum* species complex (FTSC)) may be involved. Botha et al. (2014) studied the *Fusarium* present in Eastern Cape (South Africa) dairy pastures where cattle intoxication outbreaks occurred. Strains were identified based on *TEF* sequences, and similar to our survey (Dewing et al. 2025), they mostly detected FIESC species and did not detect *F.torulosum*. Both studies, Botha et al. (2014) and Dewing et al. (2025), detected *F.brevicaudatum*, *F.clavus*, *F.croceum* and the two species we describe above as *F.pascuum* and *F.mariecurieae* (Suppl. material 4). Additionally, our study identified *F.cumulatum*, *F.heslopiae*, *Fusarium*FIESC 27 and *Fusarium* sp. nov. 1, which were not detected in the study by Botha et al. (2014). Conversely, Botha et al. (2014) identified *F.tangerrinum* and *Fusarium*FIESC 34 (undescribed), which were not found in our survey.

Although the mycotoxigenic potential of the species described in this study is unknown, members of the FIESC have been reported to produce various mycotoxins (Phillips et al. 1989; Logrieco et al. 1998; Hestbjerg et al. 2002; Kosiak et al. 2005; Villani et al. 2016; O’Donnell et al. 2018). Many of these, especially deoxynivalenol, fumonisins and zearalenone, can adversely affect cattle health, leading to symptoms such as decreased conception rates, reproductive disorders, feed refusal, gastrointestinal problems, immunosuppression, reduced animal performance, tremors, weight loss and even death (Trenholm et al. 1985; European Food Safety Authority 2004; Morgavi and Riley 2007; Fink-Gremmels 2008). However, toxicological information and their effects on animals for some commonly produced secondary metabolites, such as beauvericin, remain unavailable (de Felice et al. 2023; Hasuda and Bracarense 2024). This lack of information is often due to the absence of regulations for these mycotoxins, resulting in a lack of standardised testing methods, as well as limited monitoring and reporting requirements. It is also crucial to consider the potential synergistic, additive, or antagonistic interactions between emerging mycotoxins and other toxins in animal feed, as these combinations could pose unexpected health risks (Křížová et al. 2021). This is particularly true for FIESC that has only occasionally been linked to cattle poisoning, possibly due to a lack of sufficient studies on pasture fungal diversity. Therefore, the presence of the FIESC species in dairy pasture still poses a potential risk of mycotoxin contamination when these grasses are used for animal feed (Botha et al. 2014), and research into their mycotoxins is urgently needed.

Our study provides a valuable insight into the diversity of the FIESC in dairy pastures in the Eastern Cape. The presence of *Fusarium* species, seemingly in a consistent community in this environment, underscores the importance of further studying these species. Further research must focus on what secondary metabolites, including mycotoxins, these species produce. This will provide insights into their potential impact on cattle health in dairy pastures.

## Supplementary Material

XML Treatment for Fusarium cumulatum

XML Treatment for Fusarium goeppertmayerae

XML Treatment for Fusarium mariecurieae

XML Treatment for Fusarium pascuum

## ﻿Additional information

### Author ORCIDs

Claudette Dewing https://orcid.org/0000-0001-6208-1721

Cobus M. Visagie https://orcid.org/0000-0003-2367-5353

Emma T. Steenkamp https://orcid.org/0000-0003-0217-8219

Brenda D. Wingfield https://orcid.org/0000-0002-6189-1519

Neriman Yilmaz https://orcid.org/0000-0002-4396-4630

### Data availability

All sequence data generated for this work can be accessed via GenBank: https://www.ncbi.nlm.nih.gov/genbank/.

## Supplementary materials

Supplementary material 1 Maximum likelihood phylogenetic tree of *the Fusariumincarnatum-equiseti* species complex based on the *CaM* dataset Data type pdf Explanation note Strains of species isolated from this study are shown in black bold text; strains of newly described species are indicated in red bold text. The tree was rooted to *Fusariumconcolor*. Branch support in nodes higher than 80% are indicated at relevant branches (^T^ = ex-type, ^ET^ = epitype, ^NT^ = neotype). https://binary.pensoft.net/file/1286414This dataset is made available under the Open Database License (http://opendatacommons.org/licenses/odbl/1.0/). The Open Database License (ODbL) is a license agreement intended to allow users to freely share, modify, and use this Dataset while maintaining this same freedom for others, provided that the original source and author(s) are credited.Claudette Dewing, Cobus M. Visagie, Emma T. Steenkamp, Brenda D. Wingfield, Neriman Yilmaz

Supplementary material 2 Maximum likelihood phylogenetic tree of *the Fusariumincarnatum-equiseti* species complex based on the *RPB2* dataset Data type pdf Explanation note Strains of species isolated from this study are shown in black bold text; strains of newly described species are indicated in red bold text. The tree was rooted to *Fusariumconcolor*. Branch support in nodes higher than 80% are indicated at relevant branches (^T^ = ex-type, ^ET^ = epitype, ^NT^ = neotype). https://binary.pensoft.net/file/1286415This dataset is made available under the Open Database License (http://opendatacommons.org/licenses/odbl/1.0/). The Open Database License (ODbL) is a license agreement intended to allow users to freely share, modify, and use this Dataset while maintaining this same freedom for others, provided that the original source and author(s) are credited.Claudette Dewing, Cobus M. Visagie, Emma T. Steenkamp, Brenda D. Wingfield, Neriman Yilmaz

Supplementary material 3 Maximum likelihood phylogenetic tree of *the Fusariumincarnatum-equiseti* species complex based on the *TEF* dataset Data type pdf Explanation note Strains of species isolated from this study are shown in black bold text; strains of newly described species are indicated in red bold text. The tree was rooted to *Fusariumconcolor*. Branch support in nodes higher than 80% are indicated at relevant branches (^T^ = ex-type, ^ET^ = epitype, ^NT^ = neotype). https://binary.pensoft.net/file/1286416This dataset is made available under the Open Database License (http://opendatacommons.org/licenses/odbl/1.0/). The Open Database License (ODbL) is a license agreement intended to allow users to freely share, modify, and use this Dataset while maintaining this same freedom for others, provided that the original source and author(s) are credited.Claudette Dewing, Cobus M. Visagie, Emma T. Steenkamp, Brenda D. Wingfield, Neriman Yilmaz

Supplementary material 4 Maximum likelihood phylogenetic tree of the *Fusariumincarnatum*-equiseti species complex based on the *TEF* dataset obtained from Botha et al. (2014) and relevant reference sequences Data type pdf Explanation note Strains of species isolated from this study are shown in black bold text; strains of newly described species are indicated in red bold text. The tree was rooted to *Fusariumconcolor*. Branch support in nodes higher than 80% are indicated at relevant branches (^T^ = ex-type, ^ET^ = epitype, ^NT^ = neotype). https://binary.pensoft.net/file/1286417This dataset is made available under the Open Database License (http://opendatacommons.org/licenses/odbl/1.0/). The Open Database License (ODbL) is a license agreement intended to allow users to freely share, modify, and use this Dataset while maintaining this same freedom for others, provided that the original source and author(s) are credited.Claudette Dewing, Cobus M. Visagie, Emma T. Steenkamp, Brenda D. Wingfield, Neriman Yilmaz

Supplementary material 5 Strains examined in this study, with information about substrate, country and GenBank accessions of sequences Data type xlsx https://binary.pensoft.net/file/1286418This dataset is made available under the Open Database License (http://opendatacommons.org/licenses/odbl/1.0/). The Open Database License (ODbL) is a license agreement intended to allow users to freely share, modify, and use this Dataset while maintaining this same freedom for others, provided that the original source and author(s) are credited.Claudette Dewing, Cobus M. Visagie, Emma T. Steenkamp, Brenda D. Wingfield, Neriman Yilmaz
