# Supplementary figures and images for: ﻿Three new species of Fusarium (Nectriaceae, Hypocreales) isolated from Eastern Cape dairy pastures in South Africa
Source: MycoKeys. 2025 Mar 20;115:241–71. doi: 10.3897/mycokeys.115.148914 (PMC11950824; doi:10.3897/mycokeys.115.148914)

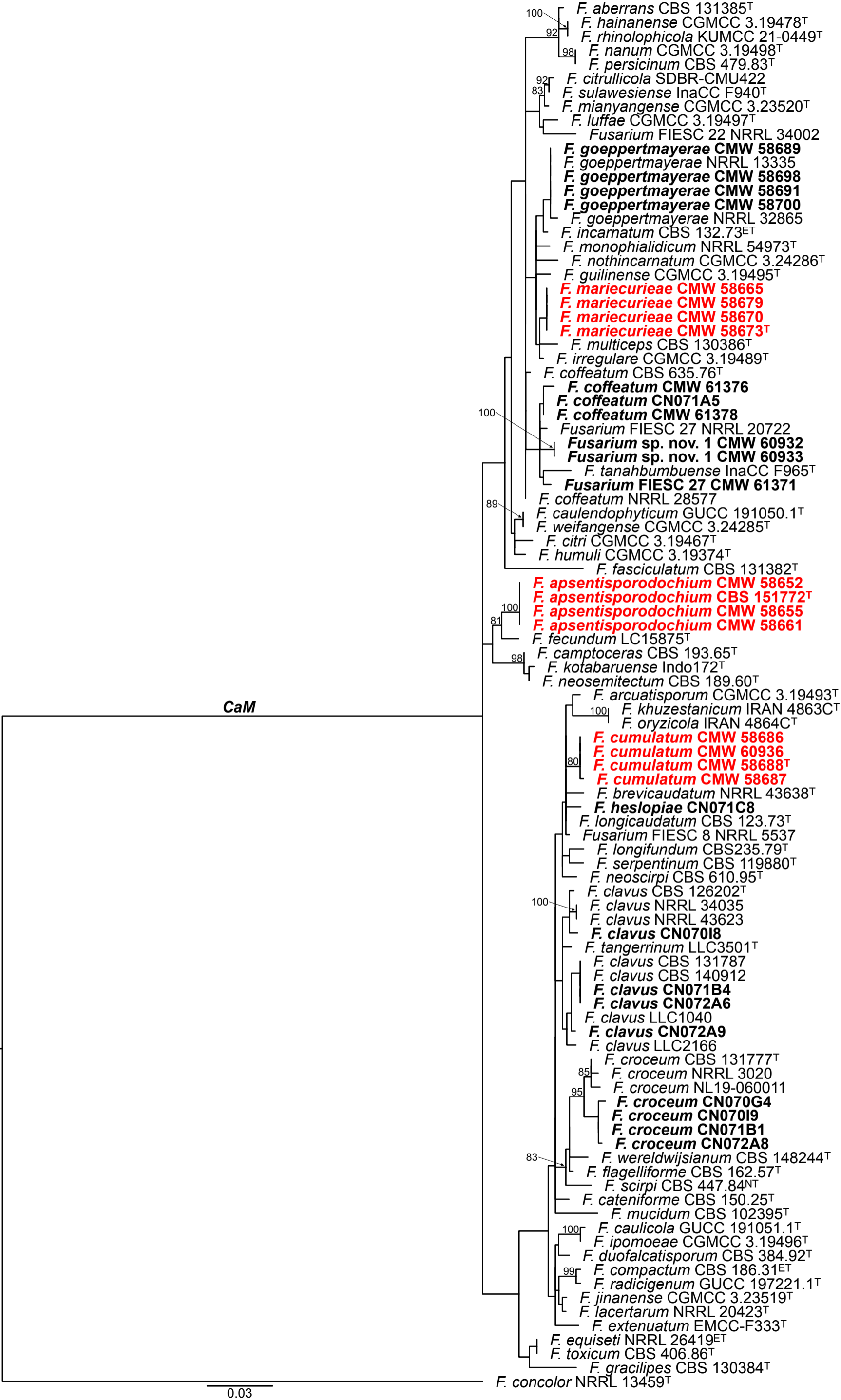

0.03

Supplement: Supplementary material 1 — Maximum likelihood phylogenetic tree of the Fusariumincarnatum-equiseti species complex based on the CaM dataset [file mycokeys-115-241-s001.pdf]

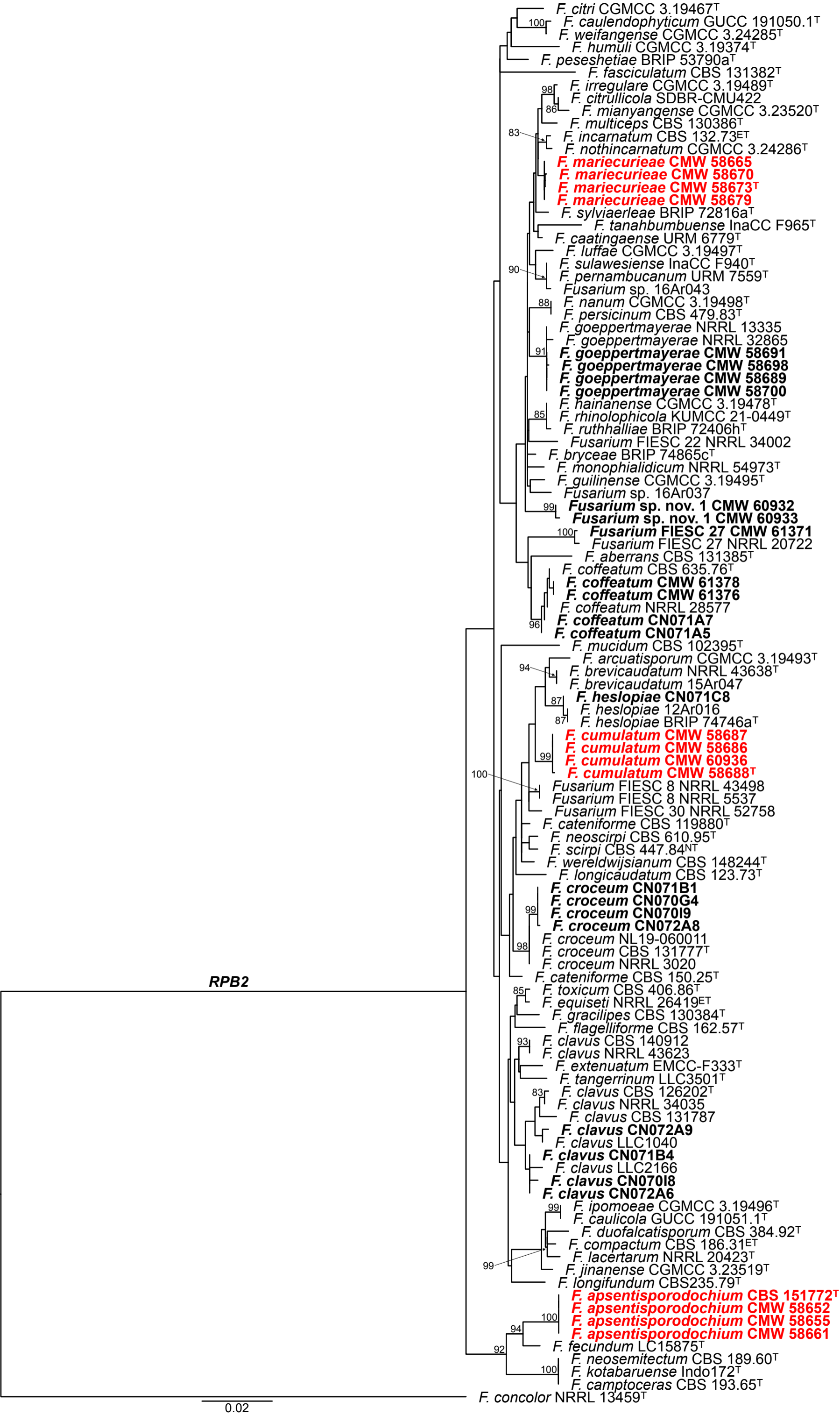

Supplement: Supplementary material 2 — Maximum likelihood phylogenetic tree of the Fusariumincarnatum-equiseti species complex based on the RPB2 dataset [file mycokeys-115-241-s002.pdf]

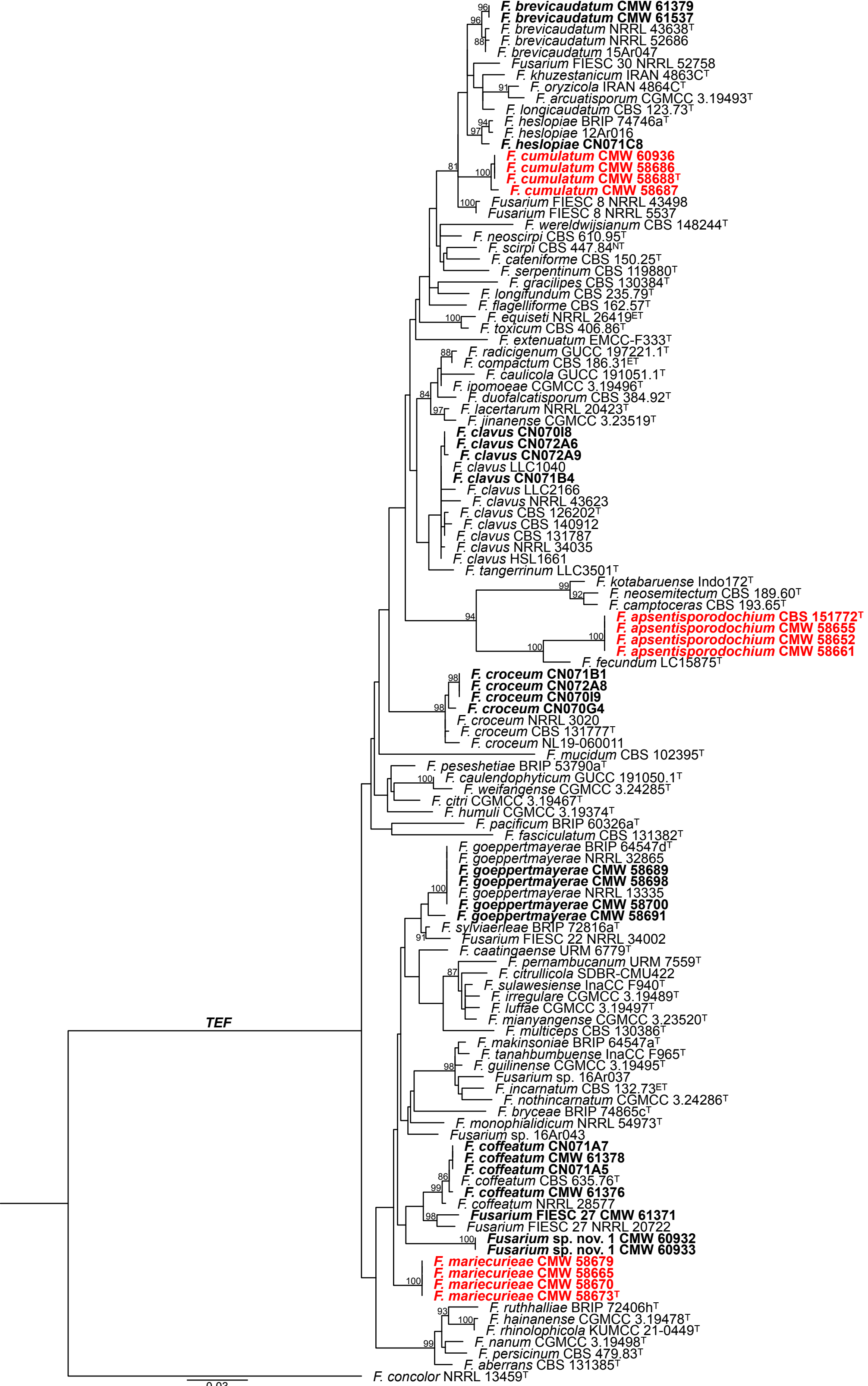

TEF

0.03

Supplement: Supplementary material 3 — Maximum likelihood phylogenetic tree of the Fusariumincarnatum-equiseti species complex based on the TEF dataset [file mycokeys-115-241-s003.pdf]
