# Supplementary material for: ﻿Three new species of Fusarium (Nectriaceae, Hypocreales) isolated from Eastern Cape dairy pastures in South Africa
Source: MycoKeys. 2025 Mar 20;115:241–71. doi: 10.3897/mycokeys.115.148914 (PMC11950824; doi:10.3897/mycokeys.115.148914)
Supplement: Supplementary material 4 — Maximum likelihood phylogenetic tree of the Fusariumincarnatum-equiseti species complex based on the TEF dataset obtained from Botha et al. (2014) and relevant reference sequences [file mycokeys-115-241-s004.pdf]

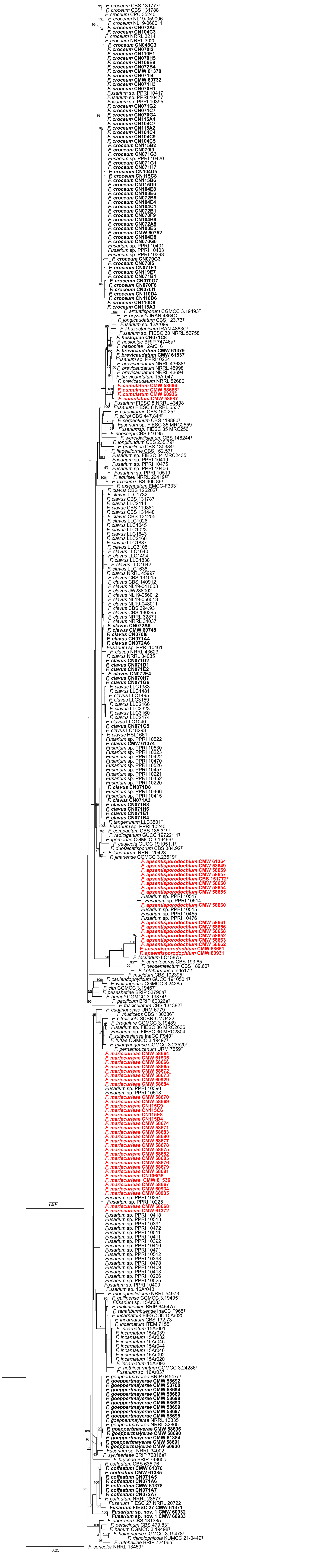
TEF
0.03

Figure S4. Maximum likelihood phylogenetic tree of the *Fusarium incarnatum*-equiseti species complex based on the *TEF* dataset obtained from Botha et al. (2014) and relevant references sequences. Strains of species isolated from this study are shown in black bold text; strains of newly described species are indicated in red bold text. Tree was rooted to *Fusarium concolor*. Branch support in nodes higher than 80% are indicated at relevant branches (^{T} = ex-type, ^{ET} = epitype, ^{NT} = neotype).

Botha CJ, Truter M, Jacobs A (2014) *Fusarium* species isolated from *Pennisetum clandestinum* collected during outbreaks of kikuyu poisoning in cattle in South Africa. Onderstepoort Journal of Veterinary Research 81: e1–e8. https://doi.org/10.4102/ojvr.v81i1.803
